# Comparison of survival times of advanced cancer patients with palliative care at home and in hospital

**DOI:** 10.1371/journal.pone.0284147

**Published:** 2023-04-13

**Authors:** Jun Hamano, Ayano Takeuchi, Masanori Mori, Yasuhiro Saitou, Takahide Yamaguchi, Nobuyuki Miyata, Masakatsu Shimizu, Ryo Yamamoto, Yousuke Kimura, Yoshiyuki Kamiyama, Yasuyuki Arai, Hiroshi Matsuo, Hideki Shishido, Kazushi Nakano, Tomohiro Nishi, Hiroka Nagaoka, Naosuke Yokomichi, Isseki Maeda, Takashi Yamaguchi, Tatsuya Morita, Takuya Shinjo

**Affiliations:** 1 Faculty of Medicine, Department of Palliative and Supportive Care, University of Tsukuba, Tsukuba, Japan; 2 Department of Preventive Medicine and Public Health, School of Medicine, Keio University, Tokyo, Japan; 3 Division of Palliative and Supportive Care, Seirei Mikatahara General Hospital, Hamamatsu, Japan; 4 GP Clinic Jiyugaoka, Tokyo, Japan; 5 Ohisama Medical Corporation, Ohisama Clinic, Hyogo, Japan; 6 Miyata Clinic, Chikusei, Ibaraki, Japan; 7 Shimizu Medical Clinic, Akashi, Hyogo, Japan; 8 Saku Central Hospital Advanced Care Center, Saku-shi, Nagano, Japan; 9 Yamato Clinic, Sakuragawa, Ibaraki, Japan; 10 Okinawa Chubu Hospital, Uruma, Okinawa, Japan; 11 Iki-iki Clinic, Yuki, Ibaraki, Japan; 12 Marguerite Clinic, Nagoya, Aichi, Japan; 13 Shishido Internal Medicine Clinic, Sakura, Chiba, Japan; 14 Nakano Zaitakuiryou Clinic, Ishiki, Kagosima, Japan; 15 Kawasaki Municipal Ida Hospital, Kawasaki, Kanagawa, Japan; 16 Department of Palliative Care, Senri-chuo Hospital, Osaka, Japan; 17 Department of Medicine, Division of Palliative Care, Konan Medical Center, Kobe, Japan; 18 Division of Palliative and Supportive Care, Palliative Care Team, and Seirei Hospice, Seirei Mikatahara General Hospital, Hamamatsu, Shizuoka, Japan; 19 Shinjo-clinic, Kobe, Hyogo, Japan; Kimura Hoospital, JAPAN

## Abstract

**Objectives:**

One primary concern about receiving care at home is that survival might be shortened because the quality and quantity of treatment provided at home will be inferior to that given in the hospital. Although our previous study demonstrated a longer survival of those with home-based palliative care (PC), it lacked adjustment for some potential confounders including symptoms and treatments during the stay. We aimed to compare the survival times among advanced cancer patients receiving home-based and hospital-based PC with adjusting for symptoms and treatments.

**Method:**

We compared survival time of participants who enrolled two multicenter, prospective cohort studies of advanced cancer patients at 45-home-based PC services between July 2017 and December 2017, and at 23-hospital-based PC services between January 2017 and December 2017. We analyzed with stratification by the estimated survival of Days, Weeks, and Months, which were defined by modified Prognosis in Palliative care Study predictor models-A. We conducted a Cox regression analysis with adjusting for potential confounders including symptoms and treatments during the stay.

**Results:**

A total of 2,998 patients were enrolled in both studies and 2,878 patients were analyzed; 988 patients receiving home-based PC and 1,890 receiving hospital-based PC. The survival time of patients receiving home-based PC was significantly longer than that of patients receiving hospital-based PC for the Days Prognosis (estimated median survival time: 10 days [95% CI 8.1–11.8] vs. 9 days [95% CI 8.3–10.4], p = 0.157), the Weeks prognosis (32 days [95% CI 28.9–35.4] vs. 22 days [95% CI 20.3–22.9], p < 0.001), and the Months Prognosis, (65 days [95% CI 58.2–73.2] vs. 32 days [95% CI 28.9–35.4], p < 0.001).

**Conclusion:**

In this cohort of advanced cancer patients with a Weeks or Months prognosis, those receiving home-based PC survived longer than those receiving hospital-based PC after adjusting for symptoms and treatments.

## Introduction

Receiving care and dying in one’s preferred place is an essential factor for a high quality of death and dying [1,2]. More than half of all people would prefer to be cared for and die at home, and the quality of death and dying are actually superior at home than in hospital [3–6]. Previous studies indicated greater pain intensity and non-home death preference predicted hospital death [7] and 68% of bereaved families in Japan preferred for the patient to die at home [8]. A qualitative study in Norway revealed that the preference to die at home was stable over time and did not change with deterioration in health status and progression in illness [9]. However, receiving care at home may be achieved only at a very late disease stage or death at home may not be achieved for multiple reasons such as insufficient health care resources, lack of caregivers, and unpreparedness of the patient and family [4,10]. One of major concern is that the quality and quantity of medical treatment provided at home will be inferior to that given in hospital and that survival might be shortened [11–13].

A small retrospective study revealed that cancer patients receiving home-based palliative care had significantly longer survival times than those in hospital, though it was lacked of adjustment for sufficient prognostic factors and was conducted at a single center with a small number of patients [14].

Then we conducted the secondary analysis of a multicenter, prospective cohort study which demonstrated that cancer patients who died receiving home-based palliative care had similar or longer survival times than cancer patients who died in hospital with specialized palliative care. However, our study did not adjust for symptom severity and medical treatments during care that would influence survival [15], whether the survival time is actually different according to the type of palliative care remains unclear. In addition, the evidence of the difference in medical treatment between palliative care at home and in hospital, which might be used to interpret the difference of survival times, is lacking. Thus, we thought it is needed to explore the difference of the survival time between advanced cancer patients receiving home-based palliative care and hospital-based palliative care with considering the symptom severity and medical treatments during care.

The present study mainly aimed to investigate the potential difference of the survival time between advanced cancer patients receiving home-based palliative care and hospital-based palliative care, adjusting not only for patient characteristics and prognostic factors, but also for symptoms and treatments.

## Materials and methods

We conducted two multicenter, prospective cohort studies of advanced cancer patients who were receiving home-based palliative care (Come Home study) or hospital-based palliative care (EASED study) in Japan, to compare the difference in survival time. Both studies aimed to document and account for the symptoms and medical treatments experienced by advanced cancer patients at the end of life. We standardized survey items and assessment tools. The Come Home study was conducted at 45 home care services between July 2017 and December 2017, and the EASED study was conducted at 23 hospital-based palliative care centers between January 2017 and December 2017. The home-based palliative care is a part of standard clinical care in Japan.

The physician primarily responsible for each patient performed an evaluation and recorded all outcome measures on the day of enrolment, and followed the patient until death or six months after enrollment. The physician evaluated the patients at least once a day in hospital, and at least once a week, and often daily, at home. The evaluation period ended when home care ended for reasons other than death or patients were discharged from hospital alive within six months. The patients in hospital-based PC usually experience an outpatient clinic, admission to a hospital, discharge to community palliative care, and die in a hospital. Both studies were conducted in accordance with the ethical standards of the Declaration of Helsinki and the ethical guidelines for research presented by the Ministry of Health, Labour, and Welfare of Japan. The institutional review boards of all participating services approved this study and main institutional review boards (Come Home study: University of Tsukuba, EASED study: Seirei Mikatahara General Hospital) approved the use of existing EASED data for secondary analysis, and to combine the data for analysis. The authors did not access to information that could identify individual participants during or after data collection.

### Patients

We enrolled eligible patients separately for both studies. The Come Home study enrolled patients when they started home-based palliative care, and the EASED study enrolled the patients when they started hospital-based palliative care at the participating facilities during the study period. The eligibility criteria for the two studies were the same; 1) 18 years old or older, 2) locally advanced or metastatic cancer (including hematopoietic neoplasms), and 3) started home-based or hospital-based palliative care at the participating facilities. Both studies excluded all patients who declined to participate in the study. Patients scheduled to be transferred or discharged within a week were excluded from receiving hospital-based palliative care.

### Outcomes

The primary endpoint of this study was survival time of participants. Survival time was defined as the period from the day of enrolment to the date of death. To adjust for background factors with a potential influence on survival time, we obtained data on the day of enrolment to formulate modified Prognosis in Palliative care Study predictor models-A (PiPs-A) [16], and data to formulate the Age-adjusted Charlson Comorbidity Index (ACCI) [17] which includes age and comorbidities. The modified PiPS-A includes the following: site of primary cancer, metastatic site, Abbreviated Mental Test score by physician rating, heart rate, anorexia, dyspnea, dysphasia, fatigue, weight loss in the previous month, Eastern Cooperative Oncology Group performance status, and global health status (rated on a specific 7-point scale used in the original study: [1] extremely poor health to [7] normal health). Symptoms were recorded as being either present or absent. Cognitive status was evaluated according to the Abbreviated Mental Test score used in the original Prognosis in Palliative Care Study models, as reported by Gwilliam et al [18]. In the current study, cognitive status was rated as absent if the score on the Abbreviated Mental Test was 4 points or more, and as present if the score was less than 3 (scoring was performed by a physician without interviewing the patient).

We also obtained data on the day of enrolment to formulate the Palliative Prognostic Index (PPI) [19] as a covariate factor of survival time. The PPI includes the following; Palliative Performance Scale (categorized into three groups: 10–20, 30–50, and 60 or more), oral intake (categorized as severely reduced, moderately reduced, or absent), edema (categorized as present or absent), dyspnea at rest (present or absent), and delirium defined by Diagnostic and Statistical Manual of Mental Disorders 5 (DSM-5) (present or absent).

We recorded several other symptoms and medical treatment factors associated with survival time based on the previous study and discussion among the researchers [20–24].

The symptoms on the day of enrolment, including pleural effusion (categorized as present or absent), asities (present or absent), bowel obstruction (present or absent), and hyperactive delirium defined by Memorial Delirium Assessment Scale item 9 (MDAS#9) [25] (present or absent) were recorded along with the symptom severity defined by the Integrated Palliative Care Outcome Scale (IPOS) and scored as 0 (not at all), 1 (slight), 2 (moderate), 3 (severe), or 4 (overwhelming), and the prevalence for any IPOS symptoms scored as 2, 3 or 4 [26,27]. We also recorded the symptoms during the enrolled periods; delirium defined by DSM-5, hyperactive delirium defined by MDAS#9, and the symptom severity at one week, three days, and one day before death (i.e., weakness or lack of energy at three days before death).

Medical treatment at the day of enrolment, such as chemotherapy within a month, use of oxygen therapy, use of any catheter, opioid dosage, and use of antipsychotic drugs, was recorded. Similarly, we recorded the medical treatment during the enrolled periods; use of antipsychotic drugs, palliative sedation, and medical treatment before death (i.e., dosage of opioids at one week before death and parenteral hydration during the 24 hours before death).

### Statistical analysis

We excluded patients whose date of death was missing, but we did not exclude or separate patients who moved from one setting to another. We conducted our analyses on complete-cases and then applied multivariate multiple imputations by chained equations (MICE) with patients having missing data before death, namely patients who moved from home to other settings, to compare survival times.

We performed MICE 10 times for the variables on the day of enrolment, i.e., use of oxygen therapy, three categories of modified PiPs-A, PPI≥6.5, presence of delirium, presence of hyperactive delirium, IPOS of dyspnea, fatigue, bowel obstruction, and for the variables during the enrolled periods, i.e., presence of delirium, presence of hyperactive delirium, palliative sedation, and use of antibiotics during the 24 hours before death. We also confirmed beforehand that the distribution and proportions of the variables on the day of enrolment did not change between subjects who were supplemented by MICE and those who were not.

To explore the difference in survival times among patients with similar background factors having a potential influence on survival time, we selected a set of confounders between the settings of PC (receiving home-based PC or hospital-based PC) and outcome (survival from the day of enrollment) based on several previous studies and clinical knowledge [20–24]. Subsequently, we compared survival times for the following modified PiPS-A survival groups: patients surviving for Days (0–13 days), Weeks (14–55 days), and Months (>55 days), based on our previous study [15]. We plotted survival curves with the Kaplan-Meier method and compared the survival times of patients who were receiving home-based palliative care and hospital-based palliative care. In addition, we conducted a Cox regression analysis to estimate the adjusted hazard ratio (HR) for survival of patients receiving home-based versus hospital-based PC by adjusting the confounders. We defined the confounders based on previous studies and discussion among the authors [19,28–33]; age (per decade), sex, primary cancer site, presence of metastasis, chemotherapy within a month, PiPs-A, PPI (≥6.5), ACCI (≥6), hyperactive delirium, use of oxygen therapy, use of any catheter, and symptoms and treatment at enrollment and during care. We defined the cutoffs of opioid dosage as greater than or equal to 120 mg/day based on a previous study that explored whether opioids influenced survival among advanced cancer patients [33].

We conducted sensitivity analyses using propensity score matching for patients’ background data of home-based and hospital-based PC to assess how substantial the effect of missing (unknown) confounders masked a true HR about the confounders of survival time. Differences in variable distributions between home-based PC and hospital-based PC were compared using Student’s t test for continuous variables and Pearson’s χ2 test or Fisher’s exact test for categorical variables. Significance was accepted at P < .05 and analyses were conducted using SPSS-J software (version 25.0; IBM, Tokyo, Japan) and SAS 9.4 (Cary, NC, USA).

## Results

In total, 2,998 patients were enrolled in both studies; 1,102 patients receiving home-based palliative care and 1,896 patients receiving hospital-based palliative care. Among them, 120 patients (home-based: 114, hospital-based:6) were excluded because the date of death was missing. Subsequently, 2,878 patients were analyzed; 988 patients receiving home-based palliative care and 1,890 patients receiving hospital-based palliative care (S1 Appendix).

The mean age was 72.5 years (95% CI: 72.1–73.0). The gastrointestinal tract/ hepatobiliary system and pancreas were the most frequent sites of primary cancer, followed by lung cancer. Almost one-third of patients had PPI ≥ 6.5, and approximately half were predicted to have a weekly prognosis by modified PiPS-A. The mean survival time was 35.2 days (95% CI: 33.6–36.7) for all patients; 51.0 days (95% CI: 47.5–54.4) for patients receiving home-based palliative care and 26.9 days (95% CI: 25.6–28.3) for patients receiving hospital-based palliative care (Table 1).

**Table 1:** Patient characteristics at enrollment.

	All patients (n = 2878)		Home-based palliative care (n = 988)		Hospital-based palliative care (n = 1890)		
	N	%	N	%	N	%	p-value
Age ≥ 65	2251	78.2	794	80.4	1457	77.1	0.046
Male sex	1518	52.7	558	56.5	960	50.8	0.004
Married	1851	64.3	701	71.0	1150	60.8	< 0.001
Live with family	2225	77.3	852	86.2	1373	72.6	< 0.001
Underage child	118	4.1	44	4.5	74	3.9	0.553
Site of primary cancer							0.057
Lung	500	17.4	182	18.4	318	16.8	
Gastrointestinal / Hepatobiliary and pancreas	1380	47.9	494	50.0	886	46.9	
Gynecological	180	6.3	61	6.2	119	6.3	
Urogenital	220	7.6	79	8.0	141	7.5	
Breast	184	6.4	53	5.4	131	6.9	
Others	414	14.4	119	12.0	295	15.6	
Metastatic site							
Anywhere	2327	80.9	724	73.3	1603	84.8	< 0.001
Liver	1066	37.0	337	34.1	729	38.6	0.023
Bone	713	24.8	213	21.6	500	26.5	0.005
Lung	981	34.1	274	27.7	707	37.4	< 0.001
Central nervous system	372	12.9	109	11.0	263	13.9	0.030
Chemotherapy within a month	370	12.9	198	20.0	172	9.1	< 0.001
Oxygen therapy	704	24.5	136	13.8	568	30.1	< 0.001
Use of any catheter	547	19.0	91	9.2	456	24.1	< 0.001
Pain IPOS*^1^ ≥ 2	1023	35.5	359	36.3	664	35.1	0.902
Shortness of breath IPOS*^1^ ≥ 2	590	20.5	210	21.3	380	20.1	0.884
Weakness or lack of energy IPOS*^1^ ≥ 2	1198	41.6	413	41.8	785	41.5	0.377
Drowsiness IPOS*^1^ ≥ 2	594	20.6	181	18.3	413	21.9	0.004
Sore or dry mouth IPOS*^1^ ≥ 2	500	17.4	139	14.1	361	19.1	< 0.001
Anorexia	2368	82.3	818	82.8	1550	82.0	0.644
Dysphagia	823	28.6	201	20.3	622	32.9	< 0.001
Weight loss in the previous month	2159	75.0	779	78.8	1380	73.0	< 0.001
Edema	1243	43.2	374	37.9	869	46.0	< 0.001
Pleural effusion	748	26.0	194	19.6	554	29.3	< 0.001
Ascites	851	29.6	284	28.7	567	30.0	0.492
Bowel obstruction	355	12.3	99	10.0	256	13.5	0.007
Delirium (DSM*^2^-Ⅴ)	677	23.5	95	9.6	582	30.8	< 0.001
Hyperactive delirium (MDAS*^3^ item 9)	367	12.8	61	6.2	306	16.2	< 0.001
Abbreviated Mental Test by physician rating ≤ 3	842	29.3	170	17.2	672	35.6	< 0.001
ECOG PS*^4^							< 0.001
0–1	129	4.5	105	10.6	24	1.3	
2	377	13.1	220	22.3	157	8.3	
3	1167	40.5	372	37.7	795	42.1	
4	1205	41.9	291	29.5	914	48.4	
Global Health							< 0.001
1: markedly poor	325	11.3	72	7.3	253	13.4	
2	746	25.9	225	22.8	521	27.6	
3	1133	39.4	373	37.8	760	40.2	
4	457	15.9	184	18.6	273	14.4	
5–7: normal health	215	7.5	134	13.6	81	4.3	
PiPs-A*^5^							< 0.001
modified PiPS-A: Months	509	17.7	258	26.1	251	13.3	
modified PiPS-A: Weeks	1428	49.6	535	54.1	893	47.2	
modified PiPS-A: Days	908	31.5	186	18.8	722	38.2	
Palliative Prognostic Index ≥ 6.5	905	31.4	167	16.9	738	39.0	< 0.001
Age-adjusted Charlson comorbidity index ≥ 6	2753	95.7	926	93.7	1827	96.7	< 0.001
Opioid dosage (OME*^6^ ≥ 120 mg/day)	284	9.9	64	6.5	220	11.6	< 0.001
Using antipsychotic drug	526	18.3	99	10.0	427	22.6	< 0.001
	Mean	95% CI	Mean	95% CI	Mean	95% CI	
Age (yrs)	72.5	72.1–73.0	72.8	72.1–73.6	72.4	71.8–72.9	0.333
Age-adjusted Charlson comorbidity index	10.8	10.7–10.9	10.5	10.3–10.6	11.0	10.8–11.1	< 0.001
Palliative Prognostic Index	5.4	5.3–5.5	4.1	3.9–4.3	6.1	5.9–6.2	< 0.001
Opioid dosage per day (OME, mg/day)	41.9	36.1–47.8	39.1	23.6–54.6	43.4	39.6–47.3	0.592

*^1^IPOS: Integrated Palliative Care Outcome Scale.

*^2^DSM: Diagnostic and Statistical Manual of Mental Disorders.

*^3^MDAS: Memorial Delirium Assessment Scale.

*^4^ECOG PS: Eastern Cooperative Oncology Group Performance Status.

*^5^PiPS-A: Prognosis in Palliative Care Study predictor model-A.

*^6^OME: Oral morphine equivalent.

### Symptoms and treatment until death

Table 2 shows the prevalence of symptoms and treatment until death. The patients with hospital-based palliative care had significantly poorer performance status health and higher symptom prevalence than patients in home-based palliative care, except for drowsiness, sore or dry mouth, hyperactive delirium at three days before death, and ascites.

**Table 2:** Symptoms and treatments until death.

	All patients (n = 2878)		Home-based palliative care (n = 988)		Hospital-based palliative care (n = 1890)		
	N	%	N	%	N	%	p-value
ECOG PS*^1^							
1 week before death							< 0.001
0–1	10	0.3	6	0.6	4	0.2	
2	76	2.6	44	4.5	32	1.7	
3	598	20.8	217	22.0	381	20.2	
4	1571	54.6	415	42.0	1156	61.2	
3 days before death							< 0.001
0–1	8	0.3	6	0.6	2	0.1	
2	20	0.7	13	1.3	7	0.4	
3	264	9.2	97	9.8	167	8.8	
4	2002	69.6	564	57.1	1438	76.1	
last day before death							0.042
0–1	5	0.2	3	0.3	2	0.1	
2	7	0.2	5	0.5	2	0.1	
3	96	3.3	28	2.8	68	3.6	
4	2200	76.4	646	65.4	1554	82.2	
Weakness or lack of energy IPOS*^2^ ≥ 2							
3 days before death	984	34.2	336	34.0	648	34.3	0.012
Drowsiness IPOS*^2^ ≥ 2							
3 days before death	703	24.4	267	27.0	436	23.1	0.356
Sore or dry mouth IPOS*^2^ ≥ 2							
3 days before death	546	19.0	186	18.8	360	19.0	0.118
Delirium (DSM*^3^-Ⅴ)							
during the care	910	31.6	211	21.4	699	37.0	< 0.001
Hyperactive delirium (MDAS*^4^ item 9)							
during the care	923	32.1	217	22.0	706	37.4	< 0.001
3 days before death	540	18.8	167	16.9	373	19.7	0.312
Antipsychotic drug for delirium							
during the care	887	30.8	112	11.3	775	41.0	< 0.001
Ascites							
3 days before death	338	11.7	97	9.8	241	12.8	0.796
Fever 1 week before death	752	26.1	131	13.3	621	32.9	< 0.001
Antibiotic use at last day before death	156	5.4	11	1.1	145	7.7	< 0.001
Parenteral hydration							
1 week before death	1046	36.3	139	14.1	907	48.0	< 0.001
last day before death	1050	36.5	131	13.3	919	48.6	< 0.001
Opioid dosage (OME*^5^ ≥ 120 mg/day)							
1 week before death	297	10.3	77	7.8	220	11.6	< 0.001
last day before death	348	12.1	87	8.8	261	13.8	< 0.001
Palliative sedation	206	7.2	45	4.6	161	8.5	0.007
	Mean	95% CI	Mean	95% CI	Mean	95% CI	
Opioid dosage per day (OME, mg/day)							
1 week before death	62.8	54.4–71.2	43.5	26.5–60.4	77.7	70.7–84.7	< 0.001
last day before death	71.1	62.6–79.7	59.5	37.6–81.3	77.1	70.8–83.5	0.128
Periods of palliative sedation (days)	1.0	0.8–1.2	0.6	0.4–0.8	3.1	2.6–3.6	< 0.001
Survival (days; median)	35.2 (21.0)	33.6–36.7	51.0 (32.0)	47.5–54.4	26.9 (17.0)	25.6–28.3	< 0.001
Good death scale (0–15)	12.0	11.9–12.2	12.2	12.0–12.4	12.0	11.8–12.1	0.048

*^1^ECOG PS: Eastern Cooperative Oncology Group Performance Status

*^2^IPOS: Integrated Palliative Care Outcome Scale.

*^3^DSM: Diagnostic and Statistical Manual of Mental Disorders

*^4^MDAS: Memorial Delirium Assessment Scale

*^5^OME: Oral morphine equivalent.

### Comparison of survival time between home-based palliative care and hospital-based palliative care

**Fig 1:**
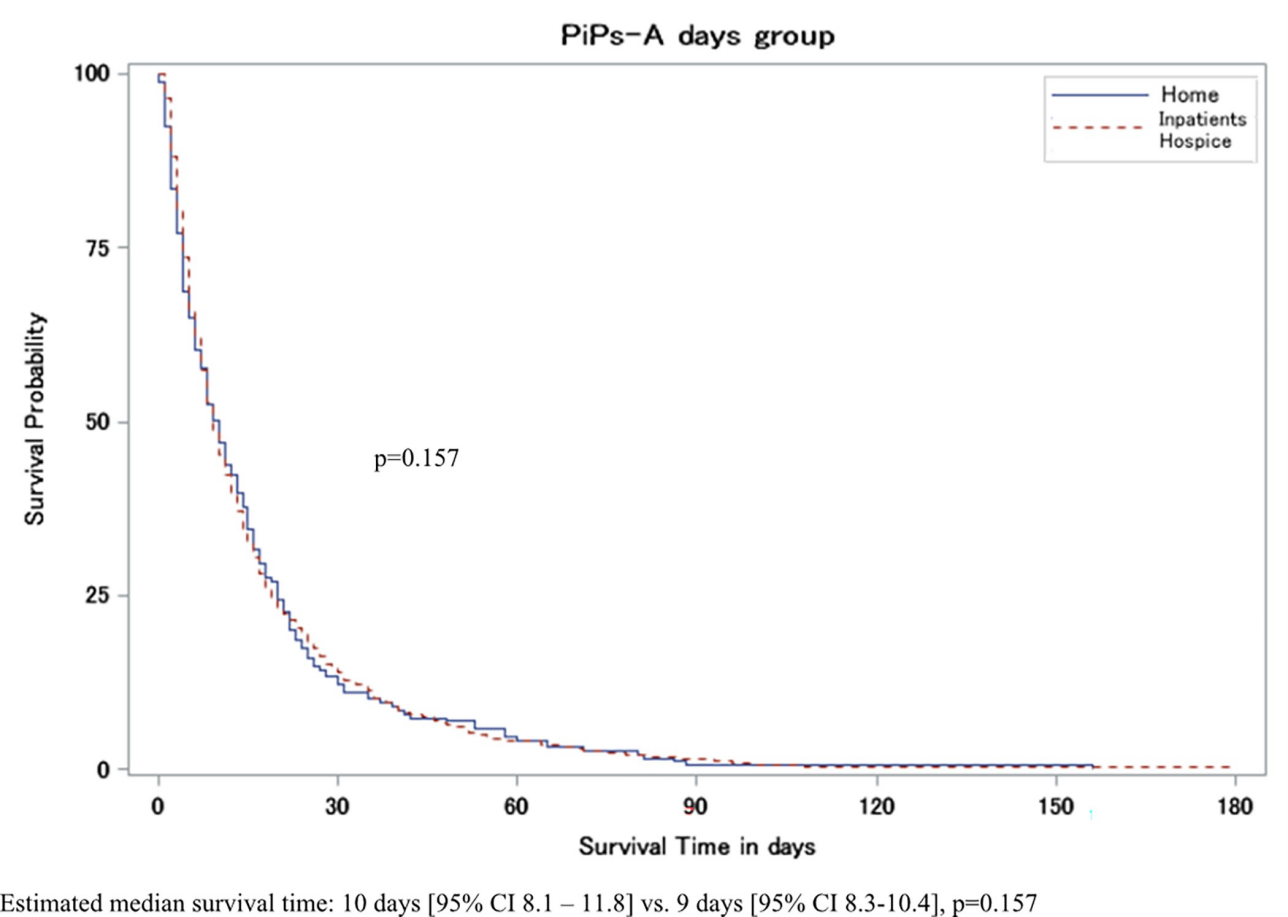
Kaplan-Meier survival curves stratified by the place of care for 3 groups defined according to Prognosis in Palliative Care Study predictor model A (PiPs-A): Days’ group (0–13 days), weeks’ group (14–55 days), and months’ group (≧56 days).

The survival of patients receiving home-based palliative care was significantly longer than that of those receiving hospital-based palliative care for the Weeks prognosis group (estimated median survival time: 32 days [95% CI 28.9–35.4] vs. 22 days [95% CI 20.3–22.9], p<0.001) and the Months prognosis group (65 days [95% CI 58.2–73.2] vs. 32 days [95% CI 28.9–35.4], p<0.001), as defined by PiPs-A. No significant difference was identified in the Days prognosis group (10 days [95% CI 8.1–11.8] vs. 9 days [95% CI 8.3–10.4], p = 0.157) (Fig 1).

Cox proportional hazards analysis revealed that home-based palliative care had a significant positive influence on survival time in both the unadjusted (HR, 0.70 [95% CI, 0.64–0.77]; P< 0.001) and adjusted models with patients’ background, symptoms, and treatment (at enrollment, during care, at 1 week before death), symptoms at 3 days before death, and treatment at last day before death (HR, 0.82 [95% CI, 0.71–0.95]; P = 0.007). High-dose opioids (oral morphine equivalent ≥ 120 mg) at one week before death had a significant positive influence on survival time, although that treatment at enrollment had a significant negative influence and that on the last day before death had no significant influence on survival time. Parental hydration at one week before death had a significant positive influence, whereas that on the last day before death had a significant negative influence (Table 3).

**Table 3:** Cox proportional hazards analysis of survival time.

	Hazard Ratio	95% CI	p-value
Unadjusted model			
Home-based	0.70	0.64–0.77	< 0.001
Adjusted model			
Home-based	0.82	0.71–0.95	0.007
Age (per decade)	0.96	0.91–1.01	0.125
Female	0.89	0.78–1.01	0.072
Site of primary cancer: Others			
Lung	1.06	0.86–1.32	0.579
Gastrointestinal	1.07	0.89–1.30	0.466
Gynecological	1.06	0.81–1.50	0.702
Urogenital	1.03	0.73–1.27	0.813
Breast	1.46	1.00–1.84	0.015
Chemotherapy within a month	0.90	0.77–1.06	0.225
Modified PiPS-A*^1^: Months			
Modified PiPS-A: Weeks	1.48	1.25–1.75	< 0.001
Modified PiPS-A: Days	3.18	2.55–3.96	< 0.001
Palliative Prognostic Index ≥ 6.5	1.37	1.16–1.62	< 0.001
Age-adjusted Charlson comorbidity index ≥ 6	0.96	0.72–1.29	0.800
Symptom and treatment at enrollment			
Pleural effusion	1.01	0.88–1.16	0.877
Ascites	1.66	1.42–1.94	< 0.001
Bowel obstruction	1.06	0.88–1.28	0.542
Pain IPOS*^2^ ≥ 2	1.08	0.90–1.15	0.242
Shortness of breath IPOS ≥ 2	1.04	0.92–1.27	0.606
Weakness or lack of energy IPOS ≥ 2	1.44	1.20–1.57	< 0.001
Drowsiness IPOS ≥ 2	1.09	0.90–1.25	0.319
Sore or dry mouth IPOS ≥ 2	1.33	1.09–1.51	< 0.001
Hyperactive delirium	1.55	1.12–2.15	0.009
Oxygen therapy	1.31	1.13–1.54	< 0.001
Use of any catheter	1.10	0.94–1.29	0.214
Opioid dosage (OME*^3^ ≥ 120 mg/day)	1.35	1.08–1.68	0.007
Using antipsychotic drug	1.07	0.91–1.25	0.416
Symptom and treatment during the care			
Delirium	0.97	0.84–1.10	0.620
Using antipsychotic drug	0.91	0.79–1.06	0.228
Palliative sedation	1.02	0.83–1.27	0.835
Symptom and treatment at 1 week before death			
Fever	0.96	0.84–1.09	0.520
Opioid dosage (OME ≥ 120 mg/day)	0.60	0.45–0.80	< 0.001
Parenteral hydration	0.81	0.66–0.99	0.040
Symptom at 3 days before death			
Weakness or lack of energy IPOS ≥ 2	1.07	0.93–1.22	0.342
Drowsiness IPOS ≥ 2	0.77	0.67–0.88	< 0.001
Sore or dry mouth IPOS ≥ 2	0.95	0.82–1.09	0.451
Ascites	0.89	0.74–1.06	0.177
Treatment at last day before death			
Opioid dosage (OME ≥ 120 mg/day)	1.11	0.86–1.43	0.437
Parenteral hydration	1.29	1.05–1.57	0.014
Antibiotic use	1.03	0.80–1.32	0.819

*^1^PiPS-A: Prognosis in Palliative Care Study predictor model-A.

*^2^IPOS: Integrated Palliative Care Outcome Scale.

*^3^OME: Oral morphine equivalent.

## Discussion

To the best of our knowledge, this is the first large-scale prospective, multicenter study to compare the survival time of advanced cancer patients receiving home-based palliative care or hospital-based palliative care adjusting for symptoms and treatment factors.

The most important finding of this study is that advanced cancer patients receiving home-based palliative care with an estimated Weeks or Months prognosis survived longer than those receiving hospital-based PC, whereas those with an estimated Days prognosis survived similar times receiving hospital-based palliative care after adjusting for symptoms and treatments. Our previous multicenter study suggested that cancer patients who died receiving home-based palliative care had similar or significantly longer survival times than those who died in hospital with specialized palliative care services, although that study lacked adjustment for symptoms and medical treatment. As several previous studies demonstrated that symptoms and treatment are associated with the survival time of advanced cancer patients [34–37], our current study was novel to present that advanced cancer patients receiving home-based palliative care with an estimated Weeks or Months prognosis survived longer than those receiving hospital-based palliative care by adjusting for symptoms and treatment factors.

One possible hypothesis that estimated Weeks or Months prognosis survived longer than those receiving hospital-based palliative care might be due to the difference in the living environment. In other words, the inpatient environment may interfere with the patient’s autonomy and motivation, which in turn may negatively affect activities, appetite, and other matters necessary for survival. Another possible hypothesis that an estimated Days prognosis had similar survival times was the physiological effects of the dying process prevail over the type of specialist palliative care. This explanation would reflect the concept of palliative care; palliative care intends neither to hasten or postpone death and focus on relief from pain and other distressing symptoms. This result implied that distinguishing the Days prognosis is important to decide treatment and care plan.

Of note, our study did not indicate a clear association between the quality of symptom control or burden of suffering and survival time. However, previous studies demonstrated that symptoms are associated with the survival time of advanced cancer patients [34–37]. Therefore, one possible explanation was that our study could not reveal symptom control details with day-by-day assessment.

A strength of this study was the large sample size that used many adjustment variables based on multiple areas including patient characteristics, prognostic factors, symptoms, and medical treatments. Since it is ethically difficult to conduct RCT, our large-scale prospective, multicenter study with multifactorial adjustments would be the high level of evidence available [38]. Another strength of our study is that missing data caused by changes in the place of care were supplemented and analyzed with multivariate multiple imputations by chained equations.

The current study also had some limitations. First, we were unable to control the general condition of the two groups at enrollment. We thought survival time cannot be compared without aligning the general condition among two groups, and it is difficult to achieve a rigorous alignment of the general conditions, even with maximum adjustment of the various prognostic markers. Therefore, our result, advanced cancer patients with a Weeks or Months prognosis, those receiving home-based palliative care survived longer than those receiving hospital-based palliative care, could be due to their better general conditions at enrollment. However, since we adjusted for the validated prognostic scales, PiPS-A and PPI, we do not believe that there was an extremely large difference in the general condition of the two groups.

Second, we were unable to adjust for residual confounding factors affecting the choice of the type of palliative care and survival time such as the preferences of patients and their families, family support, the details of dose-response treatment, and spiritual well-being.

Third, we were unable to consider the interaction of successive symptoms and medical treatments in terms of time-dependent manner. Thus, further research is needed to clarify the potential effects of symptoms and medical treatments as time-dependent confounding factors on survival time.

Fourth, sometimes a patient at home could not be assessed on a defined date such as a week before death, three days before death, or the last day before death. It is therefore possible that some of the evaluations at home were based on physicians’ estimates, which may have caused under- or overestimation of symptoms for home-based palliative care patients. Nevertheless, we believe that our large-scale, prospective, multicenter study offers the highest available level of evidence and closely maps reality.

Fifth, we did not measure the number of patients with crossover between home and hospital parts of the study. While a high percentage of crossover may affect outcomes [39], we believe it did not seriously affect this study because very few of the facilities that participated in the two studies were located in the same area, and the study periods only partially overlapped.

Thus, we could not conclude a causal relationship or clarify the scientific mechanisms between the type of palliative of care and the survival time of advanced cancer patients in this observational study. Further observational studies based on causal inference are needed to uncover whether advanced cancer patients at home shorten their survival time compared to staying in the hospital [38]. Once this becomes clear, patients, families, and providers would feel more comfortable choosing home care if they knew that advanced cancer patients receiving home palliative care would not have a shorter survival time than those receiving inpatient palliative care.

## Conclusions

In this cohort of advanced cancer patients receiving home-based palliative care with an estimated Weeks or Months prognosis survived longer than those receiving hospital-based PC, whereas those with an estimated Days prognosis survived similar times receiving hospital-based palliative care after adjusting for symptoms and treatments. Further studies are needed to clarify that the survival time of home patients is not shorter than that of inpatients.

## Supporting information

S1 Appendix Participant flow. (DOCX)
