# Supplementary material for: Comparison of survival times of advanced cancer patients with palliative care at home and in hospital
Source: PLoS One. 2023 Apr 13;18(4):e0284147. doi: 10.1371/journal.pone.0284147 (PMC10101528; doi:10.1371/journal.pone.0284147)
Supplement: S1 Appendix — (DOCX) [file pone.0284147.s001.docx]

Appendix 1 Participant flow

Enrolled

2998 patients

Hospital-based palliative care

1896 patients

Home-based palliative care

1102 patients

• Excluded due to unknown date of death

(n=120; 6 patients in PCUs and 114 patients in home care)

• No patient declined to participate in this study

Hospital-based palliative care

1890 patients

(257 patients were discharged alive)

Home-based palliative care

988 patients

(293 patients had discontinued their home care)

Analyzed

2878 patients
