# Supplementary material for: Comparison of survival times of advanced cancer patients with palliative care at home and in hospital
Source: PLoS One. 2023 Apr 13;18(4):e0284147. doi: 10.1371/journal.pone.0284147 (PMC10101528; doi:10.1371/journal.pone.0284147)
Supplement: Attachment — Submitted filename: R1_Response to Review Comments_PLOS ONE.docx [file pone.0284147.s002.docx]

Response to Review Comments

Reviewer #1:

1. From my experience, PC patients with a burden of symptoms that requires visits at home, care more about symptom-control than they care about survival time. Still it is totally acceptable to center this paper on survival time, but it would be strengthened if more comments could be added on the quality of symptom control/burden of suffering (e.g. in the abstract). In my opinion, adequate symptom control and survival time should always be linked in PC and displayed in close connection to each other.

**Reply#1 We agree with this comment. Although unfortunately, our study could not indicate a clear association between the quality of symptom control or the burden of suffering and survival time. However, we added this fact and possible explanation in the Discussion section. (p.23, l.207 - 210)**

2. The baseline data shows that the timing of enrollment in the two different types of care may differ in a clinically significant way and may therefore have a big influence on “survival time”. Table 1 shows, that patients with a “markedly poor health” are much more frequent in hospital-based PC (13.4% vs. 7.3%) which is also the case for a bad “palliative care prognostic index” (39.0% vs. 19.9%). Most of these factors are controlled for in the cox-regression model, but some are necessarily missing. I was confused though, that the ECOG wasn’t included in the model. Papers that the authors have cited (e.g. Just et al 2021) have shown, that performance status is an important factor connected to survival time. Table 2 shows clinically relevant differences in the ECOG regarding the two cohorts with a much higher burden of disability in the hospital group. I would therefore like to ask the authors to recalculate their model including ECOG upon enrollment/first available time-point as an independent variable.

**Reply#2 As the reviewer pointed out, the timing of enrollment in the two different types of care may differ. Moreover, we agree with the reviewer's comment that the performance status as the independent variable is essential. Since the PiPS-A and Palliative prognostic index include the component of performance status, we exclude the ECOG as the independent variable in terms of multicollinearity.**

3. Reasons for a decreased survival time in hospitals over time may be due to “hospitalization hazards” such as hospital acquired infections (e. g. MSRA, ESBL, Noro-Virus, SARS-CoV-2, etc.). Does the data include information on complications during the hospital stay?

**Reply#3 As the reviewer pointed out, hospital acquired infections and other adverse event in hospital might affect the survival time in hospital. Since, we did not assess the reason of the fever, it is difficult to speculate the “hospitalization hazards”. Therefore, the detailed assessment of the “hospitalization hazards” is important to conduct high quality observational study mentioned in recent article (Hernán, M. A. (2021). Methods of Public Health Research - Strengthening Causal Inference from Observational Data. The New England Journal of Medicine, 385(15), 1345–1348.** [**https://doi.org/10.1056/NEJMP2113319**](https://doi.org/10.1056/NEJMP2113319)**).**

Reviewer #2

My main concerns about the study remain the same:

The main rationale the authors have for the study is that in their previous study comparing home PC clients and hospital PC clients they did not adjust for symptom severity and medical treatments during care. In other words, they want to shift the interest from going from a description of clients of two services to exploring a causal question about whether one of the services (causally) lengthens or shortens survival. That would, after all, be the reason for controlling for potential confounders.

I think that, despite the efforts done by the authors in refining some of their analysis compared to an early version of the manuscript, it is impossible to answer this causal question with the data they have. There is so much suspicion of residual confounding and at the same time there is not really a qualitative criterion for causality (eg the Bradford-Hill criteria) that is addressed (plausibility would be a minimum). I just have too much doubt about the contribution this manuscript would make to the state of science (and would even present a misleading message).

The analysis perhaps most plausibly shows that those clients referred to home PC are different from those referred to hospital PC, not only in their symptom severity but also in several other characteristics that the study did not measure. Like in other places around the world, referrals to hospital PC most likely just happen later than referrals to home PC.

But this manuscript goes way beyond that modest claim and infers a causal effect of the type of service. The authors even claim that their study provides the best possible evidence (with an RCT being impossible). I disagree that the study does.

**Reply#1 We already mentioned what reviewer concerned in limitation section as “however, we could not conclude a causal relationship or clarify the scientific mechanisms between the type of palliative of care and the survival time of advanced cancer patients in this observational study.” (p.24, l.224 - l.226)**

**In addition, we changed the description of discussion from “our current study was novel in confirming that patients receiving home-based palliative care had longer survival times than those receiving hospital-based palliative care by adjusting for symptoms and treatment factors.” to “our current study was novel to imply that patients receiving home-based palliative care had longer survival times than those receiving hospital-based palliative care by adjusting for symptoms and treatment factors.” (p.22 l.199)**

**Furthermore, we changed the description of discussion from “our large-scale prospective, multicenter study with multifactorial adjustments is considered to be the highest level of evidence available.” to “our large-scale prospective, multicenter study with multifactorial adjustments is considered to be the high level of evidence available.** **However, RCTs in this area will continue to be ethically challenging, so it is necessary to perform the high-quality observational studies proposed in a recent article^38^.” We cited new article for this sentence. (Hernán, M. A. (2021). Methods of Public Health Research - Strengthening Causal Inference from Observational Data. The New England Journal of Medicine, 385(15), 1345–1348.** [**https://doi.org/10.1056/NEJMP2113319**](https://doi.org/10.1056/NEJMP2113319)**))**

Another comment I made in the previous reviews (as an aside) also remains: while the authors claim that all the variables controlled for in their model are confounders, treatments received during care are a mediator as they would be the result of the care service they are referred to.

**Reply#2 We already mentioned what reviewer concerned in limitation section as “First, we were unable to adjust for residual confounding factors affecting the choice of the type of palliative care and survival time such as the preferences of patients and their families, family support, the details of dose-response treatment, and spiritual well-being.” (p.23, l.221 – p.24, l.224) , and “however, we could not conclude a causal relationship or clarify the scientific mechanisms between the type of palliative of care and the survival time of advanced cancer patients in this observational study.” (p.24, l.224 - l.226)**
