# Supplementary material for: Comparison of survival times of advanced cancer patients with palliative care at home and in hospital
Source: PLoS One. 2023 Apr 13;18(4):e0284147. doi: 10.1371/journal.pone.0284147 (PMC10101528; doi:10.1371/journal.pone.0284147)
Supplement: Attachment — Submitted filename: R2_Response to Review Comments_PLOS ONE.docx [file pone.0284147.s003.docx]

Response to Review Comments

Editor Comments

1.The article is about the comparison of palliative care between at-home and in-hospital. This multi-center prospective study tried to adjust the survival time between two groups with symptoms. The manuscript is controversial among reviewers because the comparison has a risk to neglect some confounders and the disease severity of the two groups does seem not the same. I partly agree with the opinion of previous Reviewer #2 who recommended the rejection, and I think that the conclusion is too strong and the conclusion should be more modest. Please reconsider the conclusion by checking the suggestion from reviewers.

On the other hand, this manuscript is a challenging study to compare two treatment styles though not perfect in order to encourage the home-based palliative care that many patients want.

**Reply#1 We really appreciate your thoughtful comments. We totally agree with your recommendation. Then we revised the discussion and conclusion based on your comments and the comments of Reviewer #1,#3.**

Minor concerns.

1. In the abstract, “at 45 home-based PC” sounds confusing. I recommend using a 45-home-based PC “services”, “centers”, or “units” in order to distinguish from patients' numbers.

**Reply#1 We revised the abstract based on your recommendation.**

2. In the abstract, I found meaningless indention. Please delete it.

A total of 2,998 patients were enrolled in both studies and 2,878 patients were analyzed; 988 patients receiving home-based PC and 1,890 receiving hospital-based PC.

The survival time of patients receiving home-based PC was significantly longer than that of patients receiving hospital-based PC for the Days Prognosis (estimated median survival time: 10 days [95% CI 8.1 – 11.8] vs. 9 days [95% CI 8.3-10.4], p=0.157), the Weeks prognosis (32 days [95% CI 28.9 -35.4] vs. 22 days [95% CI 20.3-22.9], p < 0.001), and the Months Prognosis, (65 days [95% CI 58.2 – 73.2] vs. 32 days [95% CI 28.9-35.4], p < 0.001).

**Reply#2 We deleted the meaningless indention in the abstract based on your comments.**

3. In the Ethics statement in the submission system, please indicate the form of consent obtained (written/oral) or the reason that consent was not obtained (the data were analyzed anonymously)

**Reply#3 We rewrite the Ethics statements in the submission system as “The institutional review boards of all participating services approved this study and main institutional review boards (Come Home study: University of Tsukuba, EASED study: Seirei Mikatahara General Hospital) approved the use of existing EASED data for secondary analysis, and to combine the data for analysis. Japanese law does not require individual informed consent from participants in a non-invasive observational trial such as the present study. Therefore, we used an opt-out method rather than acquiring written or oral informed consent; all patients could receive information on the study through the instructions posted on the ward or institutional website, and they had the opportunity to decline participation.”**

4. In Figure 1, I could not read the words in the PDF file. Please check whether the high-resolution image was uploaded.

**Reply#4 We re-construct the PDF files of Figure 1.**

Reviewer #1:

1. thank you for your comments as well as the revised version. I still have one minor comment regarding the abstract (as this will be the most-read part of the paper). I would suggest to rephrase the comment section as in its current form it may be misunderstood as a generalization of the results that may not be supported by the evidence and its limitations:

current version:

Conclusion

Our study revealed that advanced cancer patients with a Weeks or Months prognosis

receiving home-based PC survived longer than those receiving hospital-based PC after

adjusting for symptoms and treatments.

Suggestion:

Conclusion

In this cohort of advanced cancer patients with a Weeks or Months prognosis, those

receiving home-based PC survived longer than those receiving hospital-based PC after

adjusting for symptoms and treatments.

**Reply#1 We really appreciate your** **specific and precise. Then, we revised the conclusion based on your recommendation. In addition, we modified the discussion section to avoid the misunderstanding of readers.**

Reviewer #3:

I agree with the comments of reviewer 1 and 2.

Essentially, patients referred to home palliative care services and hospital palliative care services are inherently different. The differences cannot be fully accounted for by statistical adjustment, due to the presence of unmeasurable confounders. While many of the comments from reviewers 1 and 2 have been addressed by including statements in the limitation section, the main text, in my opinion, still overstates the conclusions that can be reasonably drawn from this study.

**Reply#1 We really appreciate your thoughtful comments. We totally agree with your recommendation. Then we revised the discussion and conclusion based on your comments and the comments of Editor and Reviewer #1.**
